# A Novel Modified ZIF-8 Nanoparticle with Enhanced Interfacial Compatibility and Pervaporation Performance in a Mixed Matrix Membrane for De-Alcoholization in Low-Concentration Solutions

**DOI:** 10.3390/molecules29184465

**Published:** 2024-09-20

**Authors:** Yun Xiong, Yifan Shu, Niyan Deng, Xiaogang Luo, Shengpeng Liu, Xiaoyu Wu

**Affiliations:** 1 Key Laboratory for Green Chemical Process of the Ministry of Education, Hubei Key Laboratory of Novel Reactor and Green Chemical Technology, Engineering Research Center of Phosphorus Resources Development and Utilization of Ministry of Education, Wuhan Institute of Technology, Wuhan 430073, China; xiongyun@vip.163.com (Y.X.); xgluo0310@hotmail.com (X.L.); spliu@wit.edu.cn (S.L.); 2 School of Chemical Engineering and Pharmacy, Wuhan Institute of Technology, Wuhan 430073, China; syf739159@outlook.com (Y.S.); ywit126@outlook.com (N.D.)

**Keywords:** shell–ligand exchange reaction, functionalized ZIF-8 nanoparticles, mixed matrix membranes, pervaporation performance, ethanol/water separation

## Abstract

This study investigated the enhancement in bioethanol recovery from mixed matrix membranes (MMMs) by functionalizing zeolite framework-8 (ZIF-8) with imidazolate. This study focused on the separation of ethanol from low-concentration ethanol/water mixtures (typical post-fermentation concentrations of 5–10 wt%). Specifically, ZIF-8 was modified by the shell–ligand exchange reaction (SLER) with 5,6-dimethylbenzimidazole (DMBIM), resulting in ZIF-8-DMBIM particles with improved hydrophobicity, organophilicity, larger size, and adjustable pore size. These particles were incorporated into a PEBAX 2533 matrix to produce ZIF-8-DMBIM/PEBAX MMMs using a dilution blending method. The resulting membranes showed significant performance enhancement: 8 wt% ZIF-8-DMBIM loading achieved a total flux of 308 g/m^2^·h and a separation factor of 16.03, which was a 36.8% increase in flux and 176.4% increase in separation factor compared with the original PEBAX membrane. In addition, performance remained stable during a 130 h cycling test. These improvements are attributed to the enhanced compatibility and dispersion of ZIF-8-DMBIM in the PEBAX matrix. In conclusion, the evaluation of nanofiller content, feed concentration, operating temperature, and membrane stability confirmed that ZIF-8-DMBIM/PEBAX MMM is ideal for ethanol recovery in primary bioethanol concentration processes.

## 1. Introduction

Bioethanol, as a renewable energy source, is able to replace part of traditional petrol or diesel, which helps to mitigate global warming [1,2]. However, one of the key steps in bioethanol production is the separation of ethanol from fermentation broth, which is an energy-intensive and cost-intensive process. Therefore, finding a highly efficient and low-energy-consuming separation technology in the primary concentration step of bioethanol is essential to ensure the economic sustainability of bioethanol production [3,4,5]. As a membrane separation technology, pervaporation, with its advantages of high efficiency, low energy consumption, and environmental friendliness, is gradually replacing traditional distillation technology as an important candidate for ethanol separation. Pervaporation is not only able to break the ethanol/water azeotropic point (91 wt% ethanol) to produce high-concentration ethanol directly, but it is also able to process low-concentration ethanol/water mixtures effectively, thus reducing the energy required for subsequent distillation [6,7,8].

In the actual bioethanol production process, the concentration of ethanol after fermentation is usually low, generally between 5 and 10 wt%. This phenomenon is mainly attributed to the limited tolerance of fermenting microorganisms (e.g., yeast) to high ethanol concentrations [9]. Therefore, 5 wt% ethanol was used as the feed solution in this study to simulate the actual conditions of the post-fermentation liquid. Although this concentration is well below the ethanol/water azeotropic point, it represents the ethanol content commonly found after fermentation in bioethanol production and is consistent with the primary separation stage of the process [10]. Therefore, the aim of our study was to create and evaluate the separation performance of different membrane materials in low-concentration ethanol solutions by the pervaporation test in order to optimize the critical primary concentration step of the process.

Depending on the materials they are composed of pervaporation membranes can be categorized as either organic or inorganic. Inorganic membranes are known for their outstanding chemical and physical properties, extensive tunability, and the potential for reuse. Nevertheless, the scope of their applications is limited by their disadvantageous costly production expenses and operating complexity [11]. However, they are typically less stable in terms of operational stability. Therefore, it is important to modify polymeric membranes suitably to enhance the operational stability of the membranes.

MMMs are a special type of membrane typically used in gas separation and liquid separation processes. These membrane structures are composed of two or more distinct kinds of material mixed together, often an organic polymer matrix and inorganic particles such as oxide or metal–organic frameworks (MOFs). The structure of MMMs enables the optimal use of the selectivity of inorganic materials and the flexibility of organic materials for separation [8,12,13,14]. However, the formation of MMMs by combinations between organic polymers and inorganic fillers to achieve specific and superior separations often requires directional modification of inorganic filler particles, as well as a reduction in interfacial defects present in MMMs [15]. Consequently, creating defect-free MMMs continues to encounter substantial challenges [16,17,18]. Multiple strategies can be used to eradicate interfacial defects present in MMMs, including grafting, crosslinking, and polymer copolymerization [19]. Additionally, the size of MOF particles may change [20], and the functional groups or ligands on their surfaces may be altered [21,22,23]. Other effective methods involve eliminating membrane surface flaws and optimizing the fabrication strategy [24]. Besides these methods, the SLER strategy plays a crucial role in altering the initial ligands to change the properties of the MOF [25,26]. For example, bulky linkers or long-chain linkers are often used to modify MOF materials efficiently, which not only makes MOF nanofillers hydrophobic but also facilitates the interaction between the nanofillers and the polymer matrix to some extent [27,28,29].

ZIF-8, as a typical representative of zeolitic imidazolate framework (ZIF) materials, not only has the advantages of thermal stability, size tunability, high specific surface area, high porosity, and cage-shaped pores, but it is also hydrophobic [30]. For MOF MMMs, the hydrophobic modification of MOF particles is essential for enhancing the separation performance of pervaporation (e.g., n-butanol/water) [31]. Therefore, a proposed approach is to further increase the hydrophobic properties of ZIF-8. Moreover, PEBAX 2533, as a pro-organic and ideal organic membrane material, combines excellent physical and chemical properties, good chemical stability, and film formation. Therefore, it is believed to be of practical significance to dope modified ZIF-8 nanoparticles into the PEBAX 2533 matrix to prepare MMMs and to use them for de-alcoholization to improve their separation performance.

In recent years, the functionalized design (pre-synthetic and post-synthetic modification) using ZIF materials followed by doping into a polymer matrix has become a conventional modification method for the preparation of membrane materials. Li et al. coated ZIF-8 nanoparticles with poly(dopamine) (PDA) to form a chemically reactive surface and then modified them with silane coupling agents, i.e., PTMS and OTMS, to prepare P-ZIF-8@PDA and O-ZIF-8@PDA particles. The particles showed a significantly enhanced water contact angle (WCA) from 132.8° to 137° and 151°, respectively, and the hydrophobicity of the particles was significantly enhanced. Subsequently, the silane-modified ZIF-8 particles were doped into PDMS to prepare MMMs, which significantly enhanced the adsorption selectivity for n-butanol. The separation factor increased by 23% compared with ZIF-8/PDMS membranes [32]. Norma et al. synthesized highly hydrophobic ZIF-71(ClBr) and ZIF-71(ClBr)-SE particles using 4-bromo-5-chloroimidazole (HBrClIm) instead of the original ligand, 4,5-dichloroimidazole (dcIm); the WCA increased to 126° and 130°, which indicated that the particles were promising adsorbents for biofuel purification [33]. Especially for ZIF-90 with active groups, various modifications have been reported. Xu et al. obtained DLA-ZIF-90 particles by modifying the surface of ZIF-90 nanoparticles with dodecylamine (DLA) by the imine condensation reaction and doped them into a PDMS matrix to prepare DLA-ZIF-90/PDMS MMMs, which showed superiority to most other PDMS-based PDMS membranes in terms of de-alcoholization separation performance [34].

In this study, a novel ZIF-8-DMBIM material was synthesized by effectively introducing DMBIM to replace the original 2-MeIM ligand in the outermost layer of nanoparticles in ZIF-8. Following the functional modification, the mixed-linker ZIF material (ZIF-8-DMBIM) displayed notable improvements in its physiochemical properties. Specifically, the material displayed enhanced hydrophobicity, a larger particle size, and a controllable pore size. After that, we doped ZIF-8-DMBIM nanoparticles into the PEBAX 2533 matrix for the first time to prepare novel ZIF-8-DMBIM/PEBAX MMMs. Examinations of the chemical structures, thermal stability, morphologies, and crystal structures for ZIF nanoparticles and MMMs were carried out. Additionally, the impact of various factors, such as different nanofillers, ZIF-8-DMBIM loading, operating temperature, feed concentration, and prolonged membrane operation, on pervaporation performance was also investigated. In conclusion, after surface functionalization with DMBIM, the dispersion of ZIF-8 nanoparticles in the MMMs was significantly improved, which greatly promoted the membrane’s ethanol/water separation performance. These modifications are significant in facilitating the development of more functional, less energy-consuming, and more environmentally friendly MMMs that are suitable for optimizing the critical primary concentration step in bioethanol production. This is also the first time that ZIF-8-DMBIM was doped with organophilic PEBAX 2533 to prepare an MMM with the ability to separate ethanol from aqueous solutions at low concentrations.

## 2. Results and Discussion

### 2.1. Characterization of ZIF Particles

**Figure 1:**
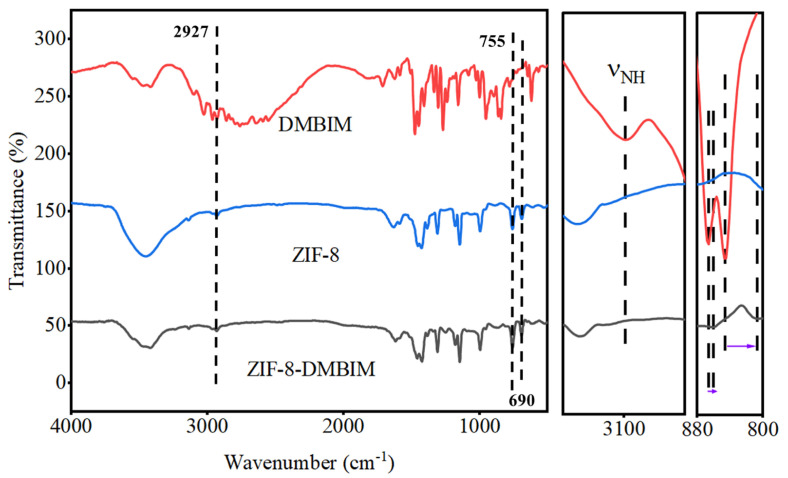
FTIR spectra of DMBIM particles, ZIF-8 particles, and ZIF-8-DMBIM particles.

The FTIR spectra of the as-prepared ZIF-8, DMBIM, and ZIF-8-DMBIM are shown in Figure 1. The spectra showed peaks at 2927, 755, and 690 cm^−1^ due to the distinctive groups in ZIF-8 [31], thus confirming the successful fabrication of ZIF-8. It was discovered that ZIF-8-DMBIM’s FTIR spectra were mostly compatible with those of ZIF-8 particles, by comparing them to those of ZIF-8, indicating that most of the groups with the structure of ZIF-8 were still retained after SLER. Moreover, there were two extra peaks observed at 813 cm^−1^ and 854 cm^−1^, which were linked to the C-H out-of-plane deformation vibration on the DMBIM benzene ring. Compared with pure DMBIM, a considerable redshift was observed from the magnification on the right, which implied that DMBIM underwent deprotonation and the nitrogen on the imidazole ring underwent a coordination reaction with Zn^2+^. Moreover, further support for the deprotonation coordination mechanism was supplied by the removal of the broadband at 3100 cm^−1^ (N-H stretching vibration) [35,36].

To further validate the chemical structure and accurately quantify the amount of substituted DMBIM, Figure S1 (Supplementary Materials) shows the ^1^H NMR results. The numbers next to or on top of the peaks correspond to the characteristic protons, while the numbers at the bottom of the peaks indicate the relative integrals [37]. The peaks at 2.66 ppm and 7.35 ppm, respectively, correlated to the -CH3 and -CH- characteristic peaks in 2-MeIM [38]. The NMR signals of ZIF-8-DMBIM were ascribed to both 2-MeIM and DMBIM; therefore, the linker composition of ZIF-8-DMBIM can be determined by normalizing the peak areas to the methyl proton of 2-MeIM [39]. Theoretically, the content of DMBIM in ZIF-8-DMBIM should be 12.6 mol%, but the NMR data revealed that the experimental content of DMBIM in the ZIF-8-DMBIM hybrid linker was 10.8 mol%. This discrepancy can be explained by the competitive coordination of 2-MeIM and DMBIM with Zn^2+^ in the hybrid linker strategy [40,41]. From a thermodynamic point of view, the Zn^2+^ metal center prefers to crystallize with 2-MeIM rather than DMBIM, which allows the metal–linker coordination reaction to be kinetically controlled, thus enabling the hybrid linker strategy to better regulate the variable linker composition [36,42].

**Figure 2:**
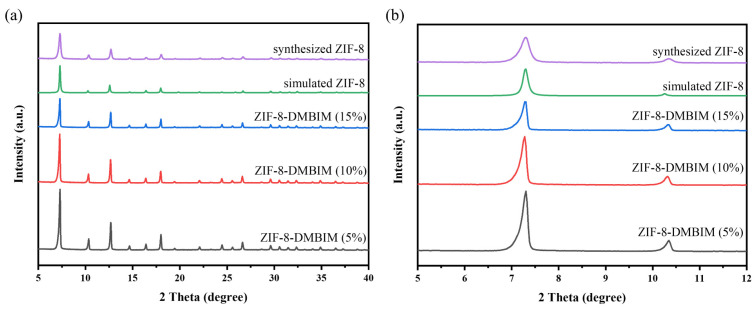
(**a**) XRD patterns of ZIF-8-DMBIM particles with different loadings. (**b**) Plot of the small-angle region, revealing minute variations in peak positions for the various structures.

The XRD spectrum diagrams of ZIF particles are shown in Figure 2a, which were used to determine whether the initial crystalline structure of ZIF-8 remained following SLER. The characteristic peaks of the prepared ZIF-8 nanoparticles matched those of the ZIF-8 nanoparticles predicted using cif (CCDC 602542) [30], demonstrating the effective fabrication of ZIF-8 nanoparticles. Furthermore, the successful partial replacement of the 2-MeIM linker in ZIF-8 with the DMBIM linker in ZIF-8-DMBIM, which effectively preserved the original crystal structure, was supported by the superior peak correspondence between ZIF-8 and ZIF-8-DMBIM [43]. However, minute variations in the parent ZIF-8 and ZIF-DMBIM’s low-angle peak locations could be seen (Figure 2b), which were brought on by the modification of the unit cell parameters [44]. The ZIF-8-DMBIM diffraction peak expanded as the quantity of DMBIM grew, showing that the manufactured crystals have a small particle size or that the test specimen is less crystalline [45]. Given this phenomenon, a Williamson–Hall plot was used to further analyze the powder XRD patterns (Figure S2) [41]. As seen in Figure S2, crystalline strain rose linearly with increasing DMBIM substitution degree. This phenomenon may be attributed to the bulky DMIBIM linker that was introduced into the ZIF-8 framework. This finding additionally provides an explanation for the peak broadening discussed before.

**Figure 3:**
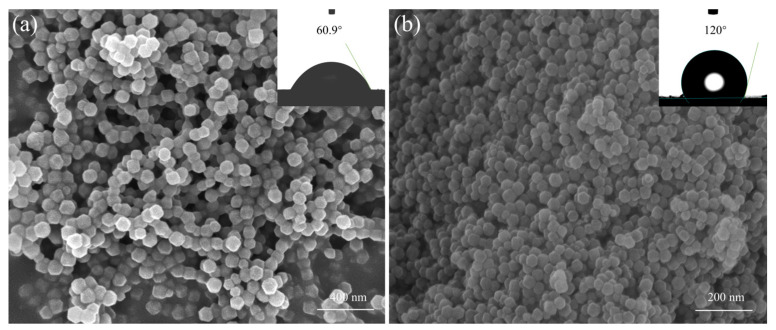
Pictures of (**a**) ZIF-8 and (**b**) ZIF-8-DMBIM nanoparticles taken with an FE-SEM. Pictures of static WCA tests that correlate to ZIF nanoparticles are shown in the insets.

Figure 3 displays the ZIF particle FE-SEM morphologies both before and after modification. The ZIF-8 nanoparticles, which had a particle size of around 20–30 nm, exhibited a typical rhombohedral crystal structure [46], as shown in Figure 3a. Though there are a few aggregated irregularly shaped particles, ZIF-8-DMBIM nanoparticles [default to ZIF-8-DMBIM (10%), which also applies below] had a morphology comparable to ZIF-8 in Figure 3b. In addition, the particle size of ZIF-8-DMBIM was significantly larger than that of ZIF-8, about 40–50 nm, which may be attributed to a small number of irregularly shaped particle aggregations. Meanwhile, it was in perfect agreement with the XRD characterization of ZIF-8-DMBIM (Figure S2), implying that replacing the original ZIF-8 ligand with DMBIM would certainly increase the nanocrystal size. In addition, a relationship between the structure and characteristics of organic linkers (such as hydrophobicity and alcoholophilicity) and particle formation might give a convincing explanation, which requires additional investigation [43]. The change in hydrophilicity after DMBIM modification was assessed by measuring the contact angle (see illustrations in Figure 3a,b). Compared with the original ZIF-8 (60.9°) [35], the WCA of ZIF-8-DMBIM was significantly increased (120.0°), which can be explained by the partial replacement of 2-MeIM by DMBIM, thus effectively increasing the volume of the linker [28]. To sum up, the two crystal structures are relatively regular, have excellent uniformity and nanoscale, and are suitable for the design of high-quality MMM materials.

As displayed in Figure S3a, the I-type adsorption isotherm increased quickly at a lower relative pressure, showing the presence of a relative amount of microporosity [47]. The modified ZIF-8-DMBIM exhibited some decrease in both specific surface area (from 1685 m^2^/g to 1241 m^2^/g) and porosity (from 0.509 cm^3^/g to 0.485 cm^3^/g, Table S2), which was in line with previous literature reports [35]. Additionally, ZIF-8-DMBIM showed no evidence of “gate opening” in the physisorption isotherms [40,43,48,49], compared to the as-prepared ZIF-8. However, the pore width distributions of synthesized ZIF-8 and ZIF-8-DMBIM (Figure S3b) displayed a major difference [36]. After SLER, the pore width distribution of ZIF-8 shifted toward larger pore widths, which considerably facilitated the passage of bigger liquid molecules like ethanol.

Figure S3c shows the TGA results. The TGA profiles of ZIF-8-DMBIM closely resembled those of the ZIF-8 nanoparticles. The curve showed slight mass loss at temperatures below 200 °C, which could be explained by the volatilization of MeOH molecules and the breakdown of the residual ligand [30,46,50]. At temperatures exceeding 600 °C, there was a significant decrease in the weight of ZIF-8 and ZIF-8-DMBIM, signifying the collapse of the crystal structure. However, ZIF-8-DMBIM remained consistently stable up to 300 °C, indicating that the integration of DMBIM took place within the original ZIF-8 structure rather than within its cavity [36]. Therefore, the ZIF-8-DMBIM particle maintained excellent thermostability.

### 2.2. Characterization of Membranes

**Figure 4:**
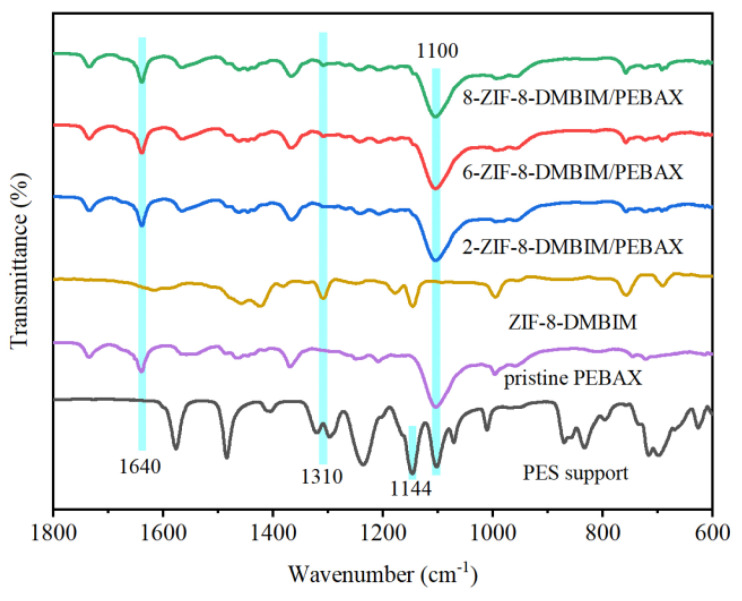
FTIR spectra of the PES support, pristine PEBAX membrane, ZIF-8-DMBIM particles, and ZIF-8-DMBIM/PEBAX MMMs with ZIF-8-DMBIM/PEBAX MMMs with various amounts of load.

The FTIR spectra of the pristine PEBAX membrane, PES support membrane, ZIF-8-DMBIM, and ZIF-8-DMBIM/PEBAX MMMs are presented in Figure 4. The chemical structure of PES mainly consisted of the following three important functional groups: benzene ring, ether group, and sulfone group. There were two characteristic peaks at 1144 cm^−1^ and 1100 cm^−1^ corresponding to the sulfone group, which was consistent with literature reports [37]. The characteristic peaks at 1100 cm^−1^ and 1640 cm^−1^ belonged to the C-O-C bond and H-N-C=O bond of the pristine PEBAX membrane, respectively [51]. The band intensity of ZIF-8-DMBIM/PEBAX MMMs at 1310 cm^−1^ (C-N bond stretching vibration) rose with the increase in ZIF-8-DMBIM loading. At the same time, ZIF-8-DMBIM/PEBAX MMMs showed no obvious band changes, revealing that the merging of the ZIF-8-DMBIM particle and PEBAX polymer matrix was merely physical.

**Figure 5:**
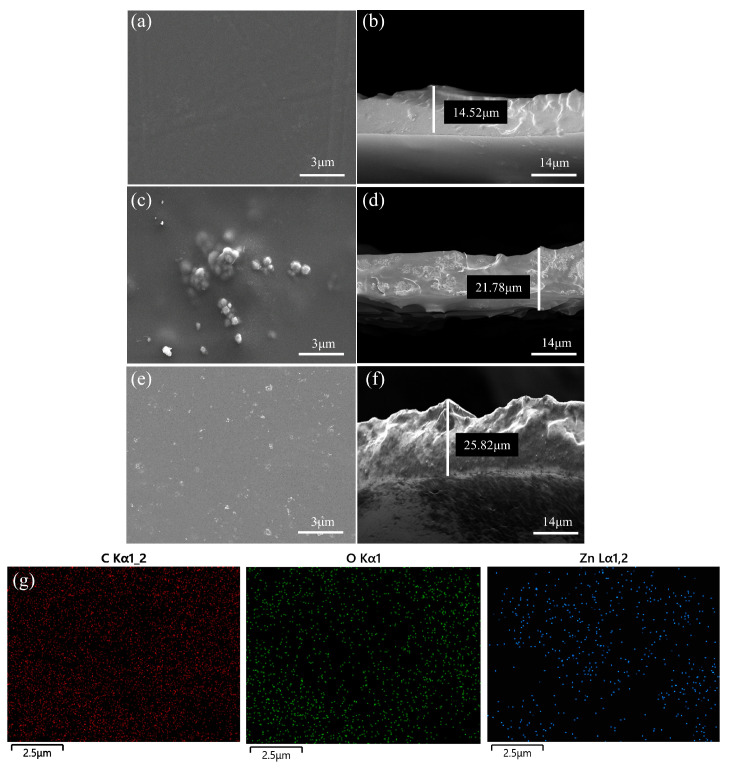
SEM pictures of the surface and cross-section of (**a**,**b**) the pristine PEBAX membrane, (**c**,**d**) the 7-ZIF-8/PEBAX MMM, and (**e**,**f**) the 8-ZIF-8-DMBIM/PEBAX MMM. (**g**) EDS mappings of 8-ZIF-the 8-DMBIM/PEBAX MMM cross-sections.

Using SEM, the surface morphologies and cross-sectional images of the original PEBAX membrane, ZIF-8/PEBAX MMM (defaults to 7-ZIF-8/PEBAX MMM, which also applies to the following sections), and 8-ZIF-8-DMBIM/PEBAX MMM were obtained (Figure 5). It was distinctly seen that the pure PEBAX membrane (Figure 5a,b) had a smooth, compact, and defect-free surface morphology. The ZIF-8 nanoparticles were embedded in the PEBAX polymer matrix with a certain amount of surface agglomeration (Figure 5c,d). However, the 8 wt% ZIF-8-DMBIM particles were tightly embedded into the PEBAX polymer matrix without obvious interfacial voids or agglomeration (Figure 5e,f). In addition, the surface of 8-ZIF-DMBIM/PEBAX MMM showed a small amount of rough micro-nanostructures consisting of nanoparticles stacked on top of each other, compared with the original PEBAX membrane, and expanded the width of the membrane, which indicated that the roughness of the MMM increased after the modification. Apart from the images of the surface morphology and cross-section, the results of the EDS mapping (Figure 5g) indicated that the ZIF-8-DMBIM particles were evenly dispersed within the PEBAX polymer matrix. In summary, after the modification of ZIF-8-DMBIM-filled particles, the surface of the PEBAX membrane was rougher, and the membrane became thicker. Meanwhile, the interfacial defects of the ZIF-8/PEBAX MMM were also improved, and there were no obvious gaps or agglomerations, which was favorable for the separation of ethanol/water.

**Figure 6:**
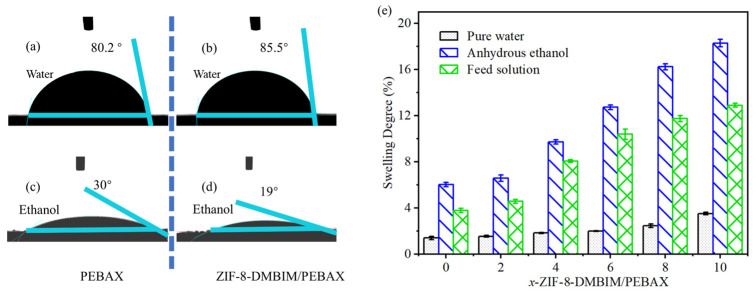
Contact angles of water and ethanol at the surface of the (**a**,**c**) PEBAX membrane and (**b**,**d**) ZIF-8-DMBIM/PEBAX MMM, respectively. (**e**) Swelling test of the x-ZIF-8-DMBIM/PEBAX MMM immersing in water, 5 wt% ethanol aqueous solution, and anhydrous ethanol.

The pervaporation performance of the membrane for de-alcoholization was related to the organophilicity and hydrophobicity of the membrane separation layer. Therefore, in this research, the hydrophobicity and organophilicity of the pristine PEBAX membrane and the ZIF-8-DMBIM/PEBAX MMMs were evaluated by the contact angles of water and ethanol, respectively. The WCA and ECA of the PEBAX membrane were 80.2° and 30° (Figure 6a,c), respectively, indicating its good affinity for ethanol. According to Figure 6b,d, ZIF-8-DMBIM/PEBAX MMM’s matching WCA and ECA were 85.5° and 19°, respectively, which might be attributed to the hydrophobicity and increased surface roughness of ZIF-8-DMBIM, demonstrating that the outstanding hydrophobicity and organophilicity of the MMM were improved by the introduction of ZIF-8-DMBIM nanoparticles.

### 2.3. Analysis of the Swelling Degree

A crucial metric for evaluating the pervaporation membrane’s affinity for the feed components was its DS. Every membrane that was involved in the experiment had a greater affinity for ethanol molecules, as seen by the DS of each membrane adhering to the order of pure water, anhydrous ethanol, and feed solution (Figure 6e). The result was comparable to the previous discussion of membrane surface features. Meanwhile, in contrast to pure water (47.8 MPa^1/2^), the Hildebrand solubility parameter of PEBAX 2533 (19.51 MPa^1/2^) [52] approximated that of anhydrous ethanol (26.5 MPa^1/2^) [53]. Typically, when two materials exhibit high solubility or compatibility with each other, their solubility parameters tend to be quite similar. This explains why PEBAX showed a greater affinity for ethanol compared with water. Consequently, the DS of the ZIF-8-DMBIM/PEBAX MMMs in water was extremely low, even displaying an unobvious trend with increasing ZIF-8-DMBIM loading. The presence and increased loading of ZIF-8-DMBIM in PEBAX caused an increase in the free volume of the membrane, which was consistent with the SEM characterization of the membranes (Figure 5b,f). Thus, the overall swelling degree of MMMs kept increasing as the loading of ZIF-8-DMBIM increased from 0% to 10%. Meanwhile, it was known that the DS of the prepared pristine PEBAX membranes (membranes loaded with zero ZIF-8-DMBIM) and the ZIF-8-DMBIM MMMs were the same as the previous statement. Both membranes barely swell or swell very little in pure water. The ZIF-8-DMBIM MMMs showed better swelling resistance in anhydrous ethanol and the feed compared with the pristine PEBAX membranes, which was due to the fact that the ZIF-8 nanoparticles were modified by the DMBIM linker into hydrophobic, organophilic nanoparticles, and doping them into PEBAX membranes allowed the MMMs to acquire such properties, resulting in excellent pure water separation performance.

### 2.4. Pervaporation Performance

**Figure 7:**
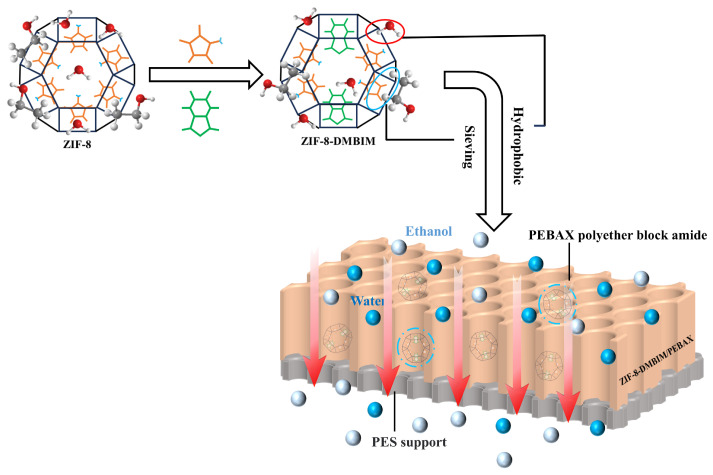
Hypothetical mechanism of the feed mixture passing through an MMM. Bluish-grey balls stand for ethanol molecules, and sky-blue balls stand for water molecules.

In this work, the pervaporation performance of MMM was relatively systematically estimated in terms of the following four aspects: different nanofillers, ZIF-8-DMBIM loading, operating temperature, and feed concentration. Figure 7 shows the possible mechanism for the separation of feed mixtures through membranes. It is noteworthy that the hydrophilic–hydrophobic interactions between the water/ethanol molecules and the membranes played a significant role in the adsorption process. In addition, the filler particles contributed to enhancing adsorption selectivity through their molecular sieving effect. Therefore, in order to achieve an improvement in the separation performance, modified ZIF-8-DMBIM nanoparticles were introduced into PEBAX in this study, as explained below.

#### 2.4.1. Impact of Different Nanofillers

**Figure 8:**
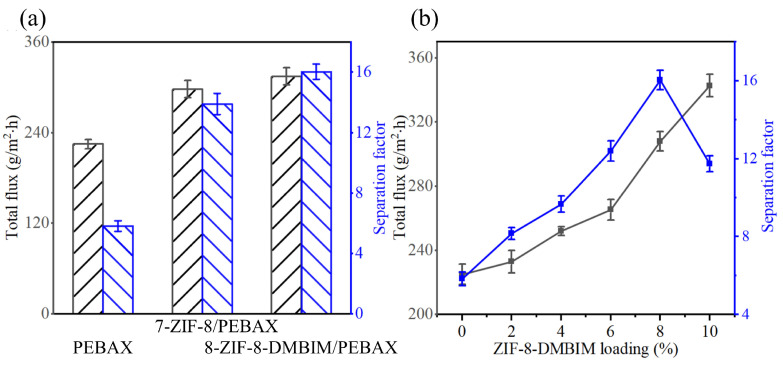
(**a**) Pervaporation performance of three kinds of PEBAX membranes with various filler particles. (**b**) Impacts of different loading on pervaporation performance of the ZIF-8-DMBIM/PEBAX MMMs.

The pervaporation performance of three distinct PEBAX membranes for the de-alcoholization of a 5 wt% ethanol/water mixed solution is depicted in Figure 8a. As shown, the total flux in the MMMs doped with ZIF-8 or ZIF-8-DMBIM exceeded that of the pristine PEBAX membrane. This enhancement is attributed to the ZIF particles’ ability to augment the free volume within the MMMs, thereby expanding the transport pathways for permeable components within the membrane [53]. Furthermore, the separation factors for all three membranes exhibited a progressive increase, likely due to the inherent hydrophobicity of ZIF-8 and the further enhancement in hydrophobicity following modification. The superior compatibility between ZIF-8-DMBIM and PEBAX also played a role in the elevated separation factor.

#### 2.4.2. Impact of ZIF-8-DMBIM Loading

The effect of ZIF-8-DMBIM loading on the separation performance of ZIF-8-DMBIM MMMs was further investigated, as shown in Figure 8b. Compared with pristine PEBAX membranes, the MMMs exhibited higher fluxes and better separation factors, which were attributed to the incorporation of porous hydrophobic ZIF-8-DMBIM particles. In addition, the flux of the MMMs consistently increased with increasing ZIF-8-DMBIM loading, which was attributed to the fact that the doping of ZIF-8-DMBIM increased the free volume of the MMMs, giving more transport paths to the permeation components and resulting in an elevated total flux. The separation factor experienced an increase and then a decrease. The organophilic property of the membrane was responsible for the increase in the separation factor, as described in the swelling analysis in Section 3.3. However, when the loading exceeded 8 wt%, because of the lack of significant chemical interaction between the modified ZIF-8-DMBIM nanoparticles and PEBAX matrix (see Section 2.2 FTIR), excessive ZIF-8-DMBIM nanoparticles produced agglomerations (Figure S4), compromising the separation factor. In conclusion, the 8-ZIF-8-DMBIM/PEBAX MMM showed the best separation performance with a maximum separation factor of 16.03 and a relative flux of 308 g/m^2^·h.

#### 2.4.3. Impact of Operating Temperature

**Figure 9:**
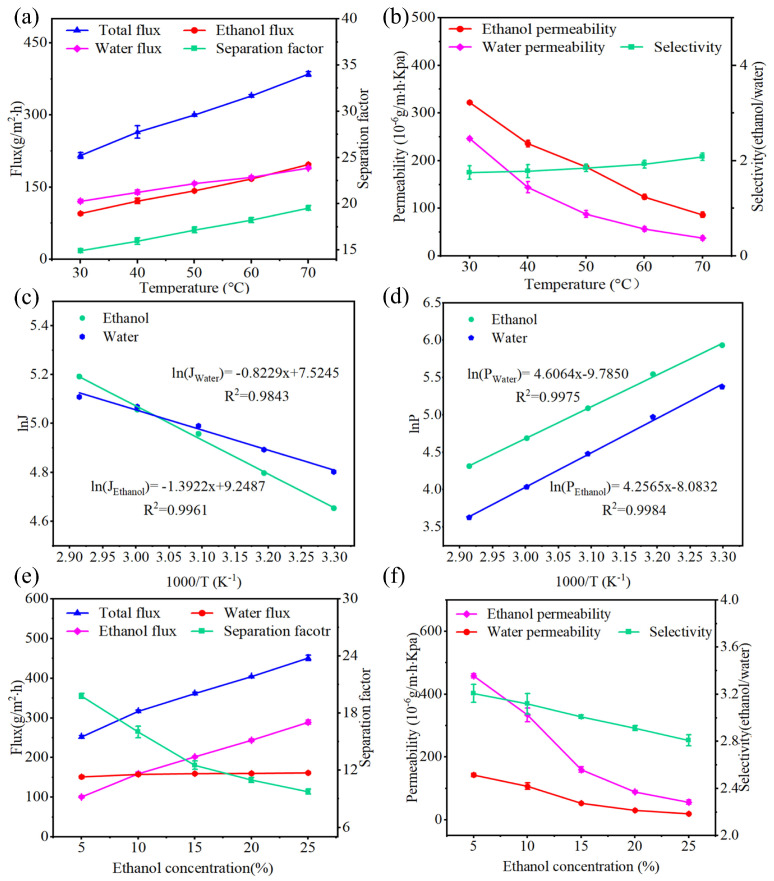
(**a**) Impact of operating temperature on the pervaporation separation performance of the 8-ZIF-8-DMBIM/PEBAX MMM: fractional flux, total flux, and separation factor; (**b**) fractional permeability and selectivity (ethanol/water); (**c**) Arrhenius curves of the flux; and (**d**) the permeability of the 8-ZIF-8-DMBIM/PEBAX MMM varying with operating temperature. Impact of the feed concentration on the pervaporation separation performance of the 8-ZIF-8-DMBIM/PEBAX MMM at an operating temperature of 50 °C: (**e**) separation factor and total and fractional fluxes and (**f**) fractional permeability and selectivity.

As mentioned above, the 8-ZIF-8-DMBIM/PEBAX MMM’s separation performance was optimal. Therefore, the 8-ZIF-8-DMBIM/PEBAX MMM was chosen in the later study to demonstrate the impact of operating conditions on the separation performance of the membrane. The pervaporation performance of the 8-ZIF-8-DMBIM/PEBAX MMM at the operating temperature ranging from 30 to 70 °C is shown in Figure 9a. It was obvious that the total and fractional flux in the membrane increased with a raised operating temperature, along with a slight increase in the separation factor. This could be attributed to the following two points: first, with the increase in the operating temperature, the saturated vapor pressure on the feed side increased, and the driving force of components through the film became larger, which accelerated the material liquid molecules through the film. Second, because of the increase in the operating temperature, the fluidity of polymer molecular chains was enhanced, and the free volume in the membrane was increased, enhancing permeation flux. It was evident that the trend in increasing ethanol flux outpaced that toward increasing water flux, which indicated that the penetration rate of ethanol molecules across the membrane was more susceptible to variations in the operating temperature, resulting in the increase in the separation factor.

To evaluate the intrinsic separation performance of the 8-ZIF-8-DMBIM/PEBAX MMM, we continued to investigate its permeability and selectivity, and the activity coefficients are shown in Table 1. With the increase in operating temperature, all permeabilities showed a downward trend and ethanol/water selectivity increased slightly (Figure 9b). At a higher operating temperature, the adsorption degree of permeable components on the membrane surface decreased, which was the primary cause of the reduction in permeability. Furthermore, the significant decline in water permeability surpassed the reduction observed in ethanol as the operating temperature increased. Consequently, as the temperature rose, so did the membrane selectivity for ethanol.

Arrhenius Equations (1) and (2) describe the associations between the operating temperature and fractional flux or permeability [31].

(1) $$J_{i}=A_{J,i}exp\left(-\frac{E_{J,i}}{RT}\right)$$

(2) $$P_{i}=A_{P,i}exp\left(-\frac{E_{J,i}}{RT}\right)\;,\;$$

where the apparent activation energy of component *i*, kJ/mol, is indicated by the symbols *E*_*J*,*i*_ and *E*_*P*,*i*_, which are derived from a slope analysis of the fitted curves for water and ethanol. The pre-exponential factor is denoted by *A*_*J*,*i*_ and *A*_*P*,*i*_, and *T* is the operating temperature, K. *R* stands for the ideal gas constant, and the value it carries is 8.314 J·mol^−1^·K^−1^.

The relationship between the fractional fluxes in the components, the fractional permeability, and the operating temperature can be determined by the Arrhenius equation, i.e., Equations (7) and (8). The results are shown in Figure 9c,d The apparent activation energy of the ethanol flux (*E*_*J*,*Ethanol*_ = 11.57 kJ/mol) was calculated to be greater than that of the water flux (*E*_*J*,*Water*_ = 6.84 kJ/mol). This implies that the ethanol molecules are more sensitive to the operating temperature; thus, the increase in ethanol flux with increasing temperature was greater than that of water, resulting in an increase in the separation factor, which is in agreement with the previous inference on the variation in the separation factor with operating temperature. In addition, the apparent activation energy of ethanol permeation (*E*_*P*,*Ethanol*_ = −35.39 kJ/mol) was also higher than that of water permeation (*E*_*P*,*Water*_ = −38.30 kJ/mol), and both were negative, indicating that the permeability of the ZIF-8-DMBIM/PEBAX hybrid matrix membrane decreased with increasing temperature. However, the decrease in ethanol permeation was smaller than that of water, thus increasing the ethanol/water selectivity [54].

#### 2.4.4. Impact of Feed Concentration

The impact of varying feed concentrations across a range of feed concentrations from 5 wt% to 25 wt% ethanol in an aqueous solution on the pervaporation performance of the 8-ZIF-8-DMBIM/PEBAX MMM was examined. The total flux of the MMM, as depicted in Figure 9e, exhibited a linear rise with higher ethanol concentrations in the feed solution. However, concurrently, the separation factor exhibited a gradual decline, which could be ascribed to the synergistic effect of the heightened driving forces along with membrane swelling. Moreover, it is notable that the significantly higher ethanol flux led to an increase in the total flux. The elevated ethanol concentration notably bolstered the partial pressure disparity (driving force) of ethanol across the membrane. The decrease in the separation factor could be because of the larger free volume of the membrane after solubilization, which resulted in the easier passage of water molecules with smaller kinetic diameters across the membrane, leading to a gradual decrease in the separation factor.

In order to investigate the intrinsic separation performance of the 8-ZIF-8-DMBIM/PEBAX MMM at different feed concentrations, permeability and selectivity were further investigated. As shown in Figure 9f, the fractional permeabilities in the membranes all decreased gradually with the increase in ethanol content in the feed solution. Moreover, the decrease in ethanol permeability was more significant than that of water permeability. Therefore, ethanol/water selectivity decreases with the increase in feed ethanol concentration. This phenomenon can be explained by the following two points: First, the higher the ethanol concentration in the feed, the more significant the coupling effect between ethanol and water molecules [55]. The mutual drag force between ethanol and water molecules restricts the ethanol and water molecules from crossing the MMM [31]. Second, as the ethanol content in the feed increases, the MMM expands more easily, and the free volume and mobility of the polymer chains are enhanced, which is more favorable for the transport of small-sized molecules [53] but may prolong the diffusion distance and lead to a decrease in the permeability of water and ethanol.

### 2.5. Membrane Stability Test

**Figure 10:**
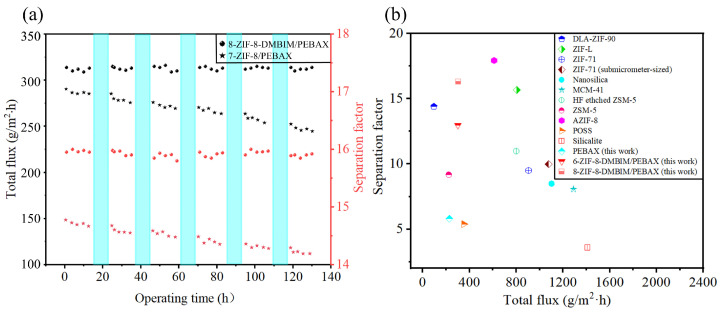
(**a**) Long-term stability test of the 7-ZIF-8/PEBAX MMM and 8-ZIF-8-DMBIM/PEBAX MMM for pervaporation de-alcoholization. (**b**) Comparison of the pervaporation performance of different MMMs in de-alcoholization.

Since the feed circulation and vacuum pump would inevitably need to be stopped during practical use, once the pump is suddenly turned on, the soaring pressures from the air hammer effect might destroy the integrity of the membrane, rendering the long-term stability performance of the membrane particularly vital in industrial production. The long-term stability performance of the ZIF-8-DMBIM/PEBAX MMM was investigated in a 5 wt% ethanol/water solution at 50 °C (Figure 10a). To guarantee a certain feed concentration for a given feed, a new feed solution of a specified concentration was periodically introduced into the feed tank during the experiment. Without disassembling, the acquired membrane was inserted into the membrane module in the interim. Even when the feed circulation and the vacuum pump were stopped at night, the feed solution was continuously in contact with the membrane (see the light blue section). Compared with the ZIF-8/PEBAX MMM, the total permeation flux and separation factor of the ZIF-8-DMBIM/PEBAX MMM remained quite stable over nearly 130 h of cyclic testing. This also shows the effectiveness of the DMBIM linker in modifying ZIF-8. Because of the linker modification, the interfacial defects of the ZIF-8/PEBAX MMMs were optimized to a large extent, which improved the long-term operational stability and some structural properties of the membranes [56,57]. As a result, the ZIF-8-DMBIM/PEBAX MMMs have the potential to be recycled and exhibit the long-term stable performance of pervaporation de-alcoholization in practical applications.

### 2.6. Performance Benchmarking

To remove ethanol from an ethanol aqueous solution at low concentrations, pervaporation performance has been reported in many works in the literature [31,34,53,58,59,60,61,62,63,64,65]. The operating conditions made a significant difference in the separation factor and permeation flux in the membranes. The performance comparisons performed here were based on the use of a 5 wt% aqueous ethanol solution as feed at 50 °C and ignoring other preparation conditions (e.g., polymer source, cross-linking degree, filler type, loading, and pre- and post-treatment of the membranes). According to Figure 10b, in comparison with other MMMs used for de-alcoholization, the present work prepared an 8 wt% loading of ZIF-8-DMBIM/PEABX MMM, which exhibited an appreciable permeate flux and high separation factor. Moreover, in comparison with the pristine PEBAX membranes fabricated in this study, the flux and separation factor of the ZIF-8-DMBIM/PEABX MMMs increased by 36.8% and 176.4%, respectively, which broke the trade-off effect between flux and the separation factor. The results indicated that the ZIF-8-DMBIM/PEBAX MMMs exhibited significant application potential in separating ethanol from water through pervaporation.

## 3. Materials and Methods

### 3.1. Materials

Zinc nitrate hexahydrate [Zn(NO_3_)_2_·6H_2_O, 99%], 5,6-dimethylbenzimidazole (DMBIM, 99%), and 2-methylimidazole (2-MeIM, 98%) were purchased from Aladdin, (Tokyo, Japan). Poly(ether sulfone) (PES) was purchased from Sumitomo Chemical (Tokyo, Japan). Methanol (MeOH, AR), triethylamine (TEA, AR), N, N-dimethyl formamide (DMF, AR), and n-butyl alcohol (n-Butanol, AR) were obtained from Sinopharm Chemical Reagent Co., Ltd. (Shanghai, China). Poly(ether block amide) (PEBAX, 2533) was supplied by Arkema in Lannemezan, France. A laboratory purification system was used to prepare deionized water (DI water) for the experiment. In addition, all utilized chemicals were employed straightly without pretreatment.

### 3.2. Preparation of ZIF Nanoparticles

#### 3.2.1. Preparation of ZIF-8

The synthesis of ZIF-8 nanoparticles was performed following the procedure described in a previously published paper [46]. Specifically, Zn(NO_3_)_2_·6H_2_O (3.000 g) and Mim (6.692 g) were separately dissolved in 144 mL of MeOH. The two solutions were then rapidly mixed and vigorously stirred for a duration of 1 h at a temperature of 23 ± 2 °C. After high-speed centrifugation, the resulting crystals were collected, washed with fresh MeOH, and dried under vacuum at 80 °C for 12 h. To aid in subsequent characterization, a portion of the product underwent additional vacuum drying at 150 °C for 2 h.

#### 3.2.2. Preparation of ZIF-8-DMBIM

**Figure 11:**
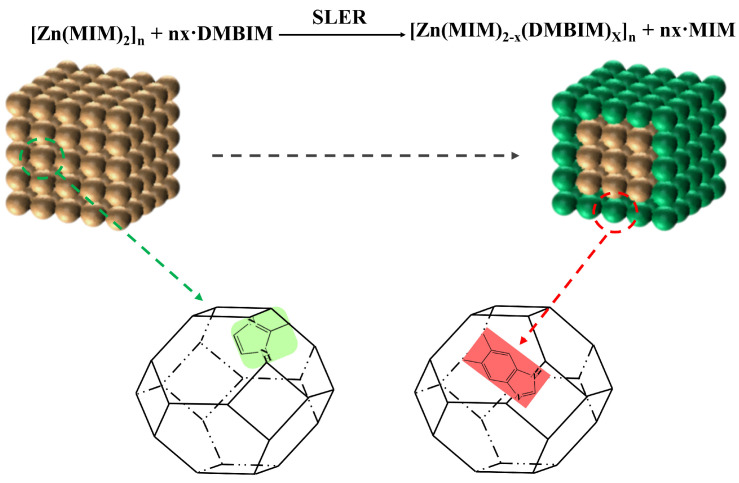
Schematic of the SLER process for ZIF-8.

Based on the synthesized ZIF-8, the synthesis of ZIF-8-DMBIM nanoparticles was carried out with the SLER method (Figure 11). The method was as follows. Newly synthesized ZIF-8 nanoparticles were dispersed in anhydrous methanol by ultrasonication until all ZIF-8 particles became invisible. DMBIM was then dissolved in anhydrous methanol with stirring until completely dissolved; thereafter, TEA was added dropwise, with ZIF-8 dispersion quickly poured into the DMBIM solution (m_ZIF-8_:m_DMBIM_:m_TEA_:m_MeOH_ = 1:1:1:200). The mixed solution was transferred to a poly(tetrafluoroethylene) container, sealed, and placed in a blast drying oven at 60 °C for 16 h. The solution that was mixed, as stated above, was then centrifuged and subsequently washed with fresh methanol. The beige ZIF-8-DMBIM obtained by centrifugation was subjected to a drying process in a vacuum drying oven at 80 °C, and a portion of it was used for subsequent membrane preparation, while the other portion continued to be activated at 150 °C for testing and characterization.

### 3.3. Preparation of the PES Supporting Membrane

The wet-phase transformation process was adopted to prepare the PES support layer. Specifically, PES was dried overnight in an oven at 120 °C; then, at 80 °C, it was dissolved in DMF to acquire a cast film solution with a mass fraction of 20 wt%, which was subject to degasification at ambient temperature. A support layer film with a thickness of about 300 μm was formed on a nonwoven fabric using the casting solution mentioned above. The freshly cast PES film was rapidly placed in a large amount of deionized water for 48 h to diffuse the DMF completely in the film. After that, the PES base film was placed in fresh deionized water to continue the diffusion. Finally, the prepared PES support layer membranes were dried for at least 6 h at 120 °C.

### 3.4. Preparation of MMMs

In this work, the solution mixing method was used to fabricate MMMs. In order to prepare ZIF-8/PEBAX and ZIF-8-DMBIM/PEBAX, a certain amount of PEBAX 2533 was added to a n-Butanol solution and dissolved with stirring at 80 °C for at least 8 h to obtain 8 wt% of PEBAX 2533 cast film solution. After that, a certain amount of ZIF particles was added to the PEBAX 2533 n-Butanol solution, ultrasonically dispersed for at least 1 h, and then stirred overnight and left to degas the mixed cast film solution. After degassing, the solution was cast onto the PES support layer using a casting knife with a thickness of 300 μm, and the thickness of the membrane (excluding the PES support layer membrane) was controlled to be approximately 30–40 μm. The produced ZIF-8/PEBAX and ZIF-8-DMBIM/PEBAX MMMs were dried for 12 h at ambient temperature, followed by an additional 12 h of heating at 50 °C for further solidification. The membranes that were created were named “x-ZIF-8/PEBAX” (in this article, ZIF-8/PEBAX refers to 7-ZIF-8/PEBAX) and “y-ZIF-8-DMBIM/PEBAX”, correspondingly, with x and y representing the ZIF particles’ mass percentages in PEBAX. The detailed doped compositions of various ZIF/PEBAX MMMs are listed in Table S1 (Supplementary Materials).

### 3.5. Swelling Experiment

In the swelling experiments, the mixed solution was poured into Petri dishes to prepare unsupported membranes. After being left at room temperature for an appropriate time, the unsupported membranes were dried under vacuum at 50 °C for a minimum of 12 h. Subsequently, various dry membrane samples were submerged at 30 °C for an amount of time in pure water, anhydrous ethanol, and feed solutions (5 wt% ethanol/water) until the weight remained constant. With absorbent paper, the liquid that was still on the membrane’s outside was removed. Three measurements were taken of each sample, and the average result was noted. To determine the percentage of the degree of swelling (DS), Equation (3) was used to determine its value:

(3) $$DS(\%)=\frac{W_{W}-W_{d}}{W_{d}}\times 100\%$$

where the weights of the wet and dry membranes are *W_w_* and *W_d_*, respectively.

### 3.6. Pervaporation Test

**Figure 12:**
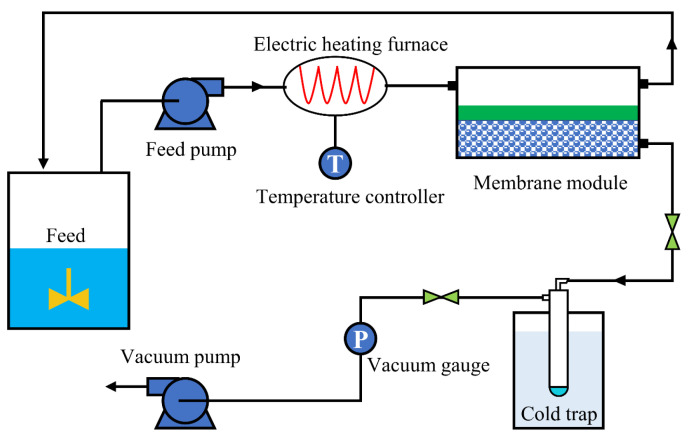
Schematic diagram of pervaporation device.

Using a separate design unit (Figure 12), the pervaporation property of the fabricated membranes was assessed. The MMMs in the test had an effective area of 19.5 cm^2^. A specific concentration of feed solution was prepared for the pervaporation test. Downstream, a vacuum pressure of 1000 Pa was maintained while the feed solution flowed through the upstream chamber of the membrane module at approximately 1.8 mL/min. The temperature operating range was adjusted, covering temperatures from 30 °C to 70 °C, using the electric heating furnace as the controller. After going through stabilization for approximately two hours, permeable components were collected hourly until the adsorption swelling equilibrium was reached. The permeate for each test was taken from the cold trap and suspended in a 55/45 volume fraction ethylene glycol aqueous solution. The concentrations of water and ethanol in the feed and permeate were measured separately using the GC (FL9790).

By using Equations (4) and (5), it was possible to determine the total flux (*J*, g/m^2^·h) and separation factor (*α*):

(4) $$J=\frac{m}{A\times t}$$

where *A* (m^2^) denotes the effective membrane area, *t* (h) indicates the operation period, and *m* (g) represents the total weight of the permeate.

(5) β=yeywxexw 

where the weight percentages of water and ethanol upstream and downstream, respectively, are *x* and *y*.

Permeability (*P_i_*), selectivity (*β*), and the pervaporation separation index (*PSI*) were additionally calculated through the application of Equations (6)–(8) so as to illustrate the inherent separation properties of the pervaporation membrane:

(6) $$P_{i}=\frac{J_{i}\times l}{x_{n,i}\gamma_{i}p_{i}^{sat}-y_{n,i}p^{p}}$$

(7) $$\alpha =\frac{P_{e}}{P_{w}}$$

(8) $$PSI=\left(\alpha -1\right)\;$$

where the molar flux of a single component is $J_{i}\;\left(mol/m^{2}\cdot h\right)$, and the thickness of the MMMs (excluding PES support layer membrane) is *l* (μm), which can be determined by SEM. The molar fractions of component *i* upstream and downstream of the membrane are *x*_*n*,*i*_ and *y*_*n*,*i*_, respectively. Both *γ_i_* and $P_{i}^{sat}$ are coefficients for component *i* upstream of the membrane, which are acquired using Wilson and Antoine equations with Aspen Plus V11 software [58] and are known as the activity coefficient and saturated vapor pressure, respectively. $P_{w}$ and $P_{e}$ denote the water and ethanol permeability, respectively, while $P^{p}$ stands for the pressure that exists downstream of the membrane.

### 3.7. Characterization and Testing

The chemical structures of ZIF particles and membranes were verified utilizing FTIR (NICOLET 6700, Thermo Fisher Scientific, Waltham, MA, USA). Test membranes with a thickness of less than 15 μm were conducted using ATR technology (ATR Learning Technology, Kyoto, Japan).

Using a BRUKER 400M spectrometer (Bruker, Ettlingen, Germany), ^1^H NMR spectra were taken following ZIF particles had been solved into d_4_-acetic acid (CD_3_CO_2_D).

Utilizing XRD (D8 ADVANCE, Bruker, Ettlingen, Germany), the crystal structures of the samples were determined over a range of diffraction angles from 5° to 40° with C_u_K_α_.

ZIF nanoparticles and membranes were observed for their morphologies using FE-SEM (Gemini SEM 300, ZEISS, Jena, Germany) and SEM (JSW-5510LV, JEOL, Tokyo, Japan). Liquid nitrogen breakage of the membranes was required to obtain a section.

Analysis of the ZIF nanoparticles’ BET surface areas, micropore volumes, and pore size distributions was performed by employing a specialized surface and porosity analyzer (Mike, ASAP2460, Micromeritics, Norcross, GA, USA). Each of the samples was subjected to degassing for 12 h in a vacuum at 150 °C before every test.

All of the materials were tested by employing the TGA (SDT Q600, TA Instruments, New Castle, DE, USA) in a nitrogen environment with an average heat rate of 10 °C/min from 30 °C to 900 °C.

With the Data Physics Instruments OCA20 (DataPhysics Instruments, Filderstadt, Germany), static WCA measurements were taken to analyze each sample’s hydrophilicity. Each sample’s measurements were averaged over 3 distinct points. The procedure for the Ethanol Contact Angle (ECA) was repeated with ethanol as the medium.

## 4. Conclusions

In this study, the functionalized modification of ZIF-8 was successfully achieved by employing the SLER strategy, which significantly enhanced the hydrophobicity, organophilicity, and pore size tunability of ZIF-8. By combining novel ZIF-8-DMBIM nanoparticles with a PEBAX 2533 matrix, MMMs with excellent performance were prepared. The results showed that the 8-ZIF-8-DMBIM/PEBAX MMM exhibited remarkable separation performance in the de-alcoholization of low-concentration ethanol solutions with a flux of 308 g/m^2^·h and a separation factor of 16.03. Compared with other permeable gasification membranes used for ethanol separation, the ZIF-8-DMBIM/PEBAX MMM showed a significant advantage in terms of separation efficiency, especially in the practical working conditions of treating low-concentration fermentation broths. Long-term stability tests further demonstrated that the ZIF-8-DMBIM/PEBAX MMM was able to maintain excellent performance for close to 130 h of continuous operation, validating its potential for industrial-scale applications. This study also clarified the importance of the application of the SLER strategy to improve the dispersion of ZIF-8 particles and their compatibility with the polymer matrix. This innovation provides a new direction for the development of efficient and sustainable bioethanol separation technologies. In view of the results of this study, future research should further optimize the functionalization of fillers and enhance the interaction between inorganic fillers and organic matrices to further improve the separation efficiency and long-term stability of the membranes. By further improving the dispersibility of nanoparticles and the binding of the polymer matrix, it is expected that pervaporation membrane materials suitable for a wider range of application scenarios will be developed, thus promoting the advancement of highly efficient ethanol separation processes in biomass production.

## Figures and Tables

**Table 1:** The relationships between the operating temperature or ethanol concentration and the activity coefficients of ethanol ($\gamma_{ethanol}$) and water ($\gamma_{water}$) in an ethanol/water binary solution.

Operating Temperature (°C)	Ethanol Concentration (wt%)	$\gamma_{ethanol}$	$\gamma_{water}$
30	5	3.72412	1.00611
40		3.87686	1.00641
50		3.99525	1.00668
60		4.08063	1.00693
70		4.13510	1.00716
50	5	3.99525	1.00668
	10	3.19352	1.02500
	15	2.64041	1.05312
	20	2.24555	1.08983
	25	1.95564	1.13435

## Data Availability

The original contributions presented in the study are included in the article/Supplementary Material, further inquiries can be directed to the corresponding author.

## Supplementary Materials

The following supporting information can be downloaded at https://www.mdpi.com/article/10.3390/molecules29184465/s1: Figure S1: The ^1^H NMR spectra of ZIF-8 particles and ZIF-8-DMBIM particles; Figure S2: Williamson–Hall analysis of the as-prepared ZIF-8 and ZIF-8-DMBIM nanoparticles; Figure S3: (a) Nitrogen adsorption and desorption isotherms, (b) pore size analysis of ZIF-8 and ZIF-8-DMBIM, and (c) TGA curves of ZIF-8 and ZIF-8-DMBIM; Figure S4: Surface and cross-section SEM images of the (a) 8% ZIF-8-DMBIM/PEBAX MMM and (b) 10% ZIF-8-DMBIM/PEBAX MMM; Table S1: The detailed doped compositions of various ZIF/DMBIM MMMs; Table S2: The BET surface area and micropore volume of different ZIF particles.
