# Supplementary material for: A Novel Modified ZIF-8 Nanoparticle with Enhanced Interfacial Compatibility and Pervaporation Performance in a Mixed Matrix Membrane for De-Alcoholization in Low-Concentration Solutions
Source: Molecules. 2024 Sep 20;29(18):4465. doi: 10.3390/molecules29184465 (PMC11433669; doi:10.3390/molecules29184465)
Supplement: Supplementary file 1 [file molecules-29-04465-s001.zip › molecules-3163641-supplementary.pdf]

Supplementary material

A Novel Modified ZIF-8 Nanoparticle with Enhanced Interfacial Compatibility and Pervaporation Performance in a Mixed Matrix Membrane for De-Alcoholization in Low-Concentration Solutions

Yun Xiong [1,2,†], Yifan Shu [2,†], Niyan Deng [2], Xiaogang Luo [1,2], Shengpeng Liu [1] and Xiaoyu Wu [1,2,*]

[1] Key Laboratory for Green Chemical Process of the Ministry of Education, Hubei Key Laboratory of Novel Reactor and Green Chemical Technology, Engineering Research Center of Phosphorus Resources Development and Utilization of Ministry of Education, Wuhan Institute of Technology, Wuhan 430073, China; xiongyun@vip.163.com (Y.X.); xgluo0310@hotmail.com (X.L.); spliu@wit.edu.cn (S.L.)

[2] School of Chemical Engineering and Pharmacy, Wuhan Institute of Technology, Wuhan 430073, China; syf739159@outlook.com (Y.S.); ywit126@outlook.com (N.D.)

* Correspondence: wxy-wit@wit.edu.cn

† These authors contributed equally to this work.

Tables and Figures

Table S1. The detailed doped compositions of various ZIF/DMBIM MMMs.

Membranes	ZIF-8/g	ZIF-8-DMBIM/g	PEBAX/g	n-Butanol solution/g
PEBAX	-	-	4	46
7-ZIF-8/PEBAX	0.35	-	4	46
2-ZIF-8-DMBIM/PEBAX	-	0.01	4	46
4-ZIF-8-DMBIM/PEBAX	-	0.02	4	46
6-ZIF-8-DMBIM/PEBAX	-	0.03	4	46
8-ZIF-8-DMBIM/PEBAX	-	0.04	4	46
10-ZIF-8-DMBIM/PEBAX	-	0.05	4	46

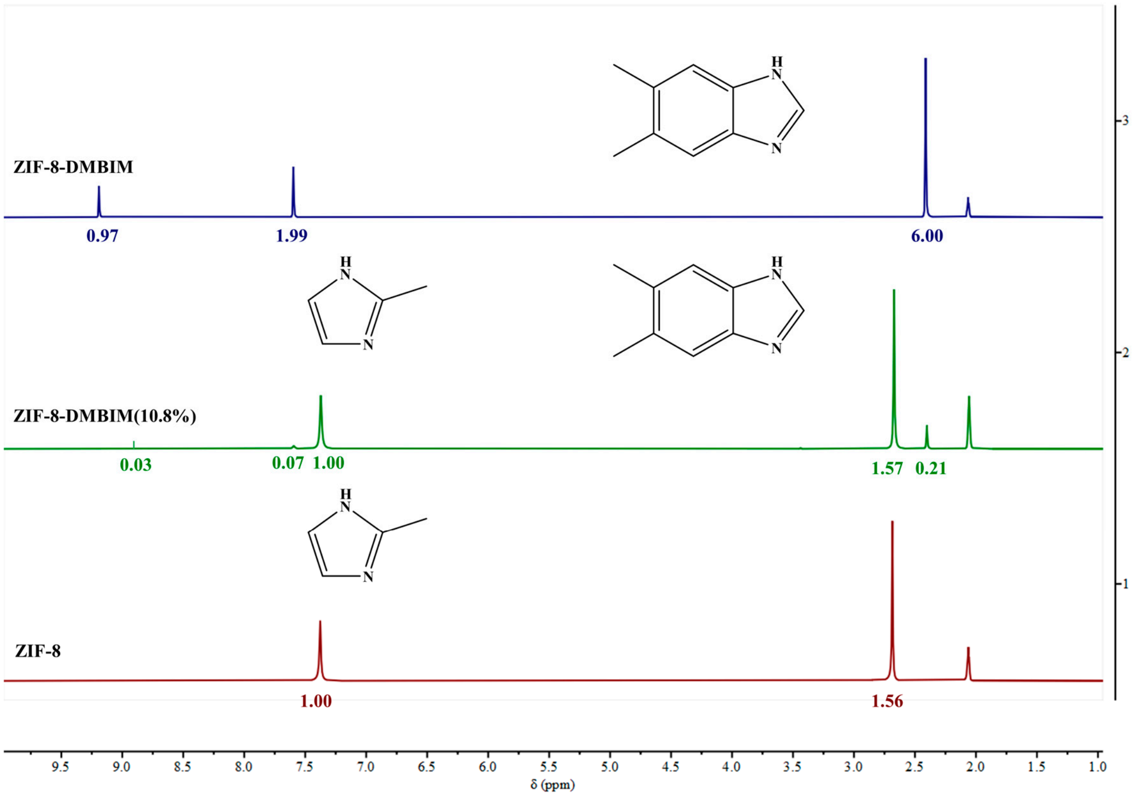

Figure S1. ^{1}H NMR spectra of ZIF-8 particle and ZIF-8-DMBIM particle.

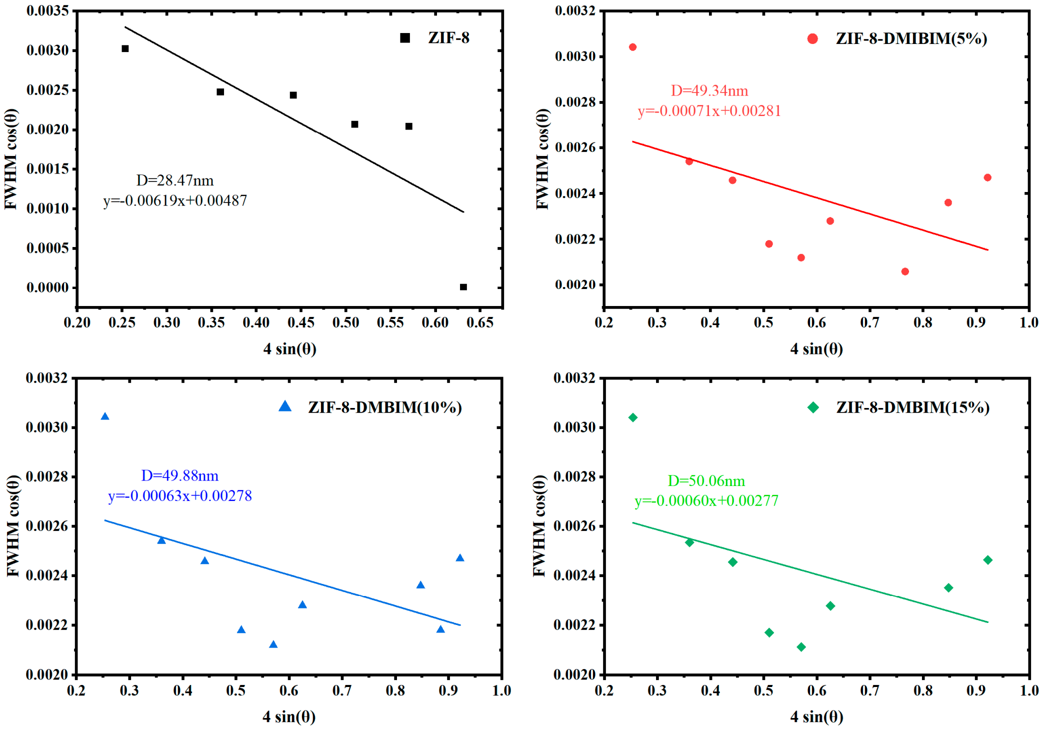

Figure S2. Williamson-Hall analysis of as-prepared ZIF-8, and ZIF-8-DMBIM nanoparticles.

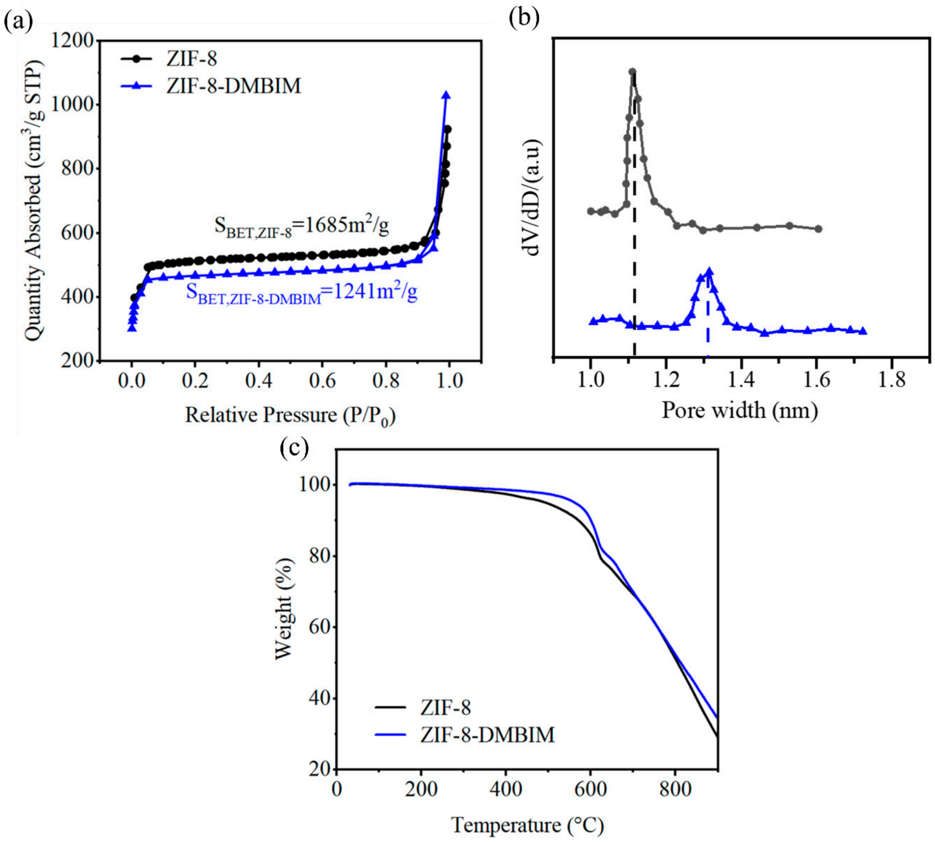

Figure S3. (a) Nitrogen adsorption and desorption isotherms, (b) pore size analysis of ZIF-8 and ZIF-8-DMBIM and (c) TGA curves of ZIF-8 and ZIF-8-DMBIM.

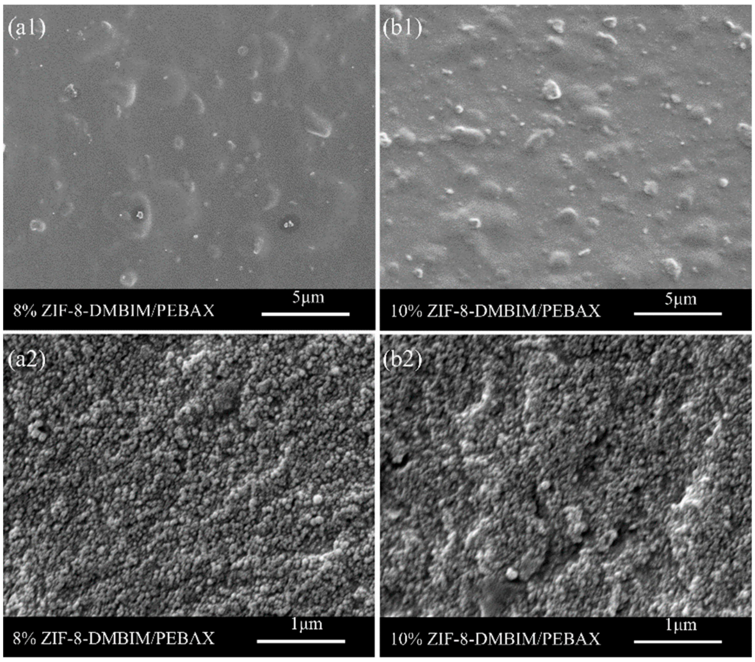

Figure S4. Surface and cross-section SEM images of (a) 8% ZIF-8-DMBIM/PEBAX MMM, (b) 10% ZIF-8-DMBIM/PEBAX MMM.

Table S2. The BET surface area, and micropore volume of different ZIF particles.

ZIF Samples	BET surface area (m^2/g)	Micropore volume (cm^3/g)
ZIF-8	1685	0.509
ZIF-8-DMBIM	1194	0.481
